# The development and validation of a radiomic nomogram for the preoperative prediction of lung adenocarcinoma

**DOI:** 10.1186/s12885-020-07017-7

**Published:** 2020-06-08

**Authors:** Qin Liu, Yan Huang, Huai Chen, Yanwen Liu, Ruihong Liang, Qingsi Zeng

**Affiliations:** Department of Radiology, The First Affiliated Hospital of Guangzhou Medical University, 151 Yanjiang Road, Guangzhou, Guangdong 510120 People’s Republic of China

**Keywords:** Lung adenocarcinoma, Computed tomography, Radiomics, Nomogram, Diagnosis

## Abstract

**Background:**

Accurate diagnosis of early lung cancer from small pulmonary nodules (SPN) is challenging in clinical setting. We aimed to develop a radiomic nomogram to differentiate lung adenocarcinoma from benign SPN.

**Methods:**

This retrospective study included a total of 210 pathologically confirmed SPN (≤ 10 mm) from 197 patients, which were randomly divided into a training dataset (*n* = 147; malignant nodules, *n* = 94) and a validation dataset (*n* = 63; malignant nodules, *n* = 39). Radiomic features were extracted from the cancerous volumes of interest on contrast-enhanced CT images. The least absolute shrinkage and selection operator (LASSO) regression was used for data dimension reduction, feature selection, and radiomic signature building. Using multivariable logistic regression analysis, a radiomic nomogram was developed incorporating the radiomic signature and the conventional CT signs observed by radiologists. Discrimination and calibration of the radiomic nomogram were evaluated.

**Results:**

The radiomic signature consisting of five radiomic features achieved an AUC of 0.853 (95% confidence interval [CI]: 0.735–0.970), accuracy of 81.0%, sensitivity of 82.9%, and specificity of 77.3%. The two conventional CT signs achieved an AUC of 0.833 (95% CI: 0.707–0.958), accuracy of 65.1%, sensitivity of 53.7%, and specificity of 86.4%. The radiomic nomogram incorporating the radiomic signature and conventional CT signs showed an improved AUC of 0.857 (95% CI: 0.723–0.991), accuracy of 84.1%, sensitivity of 85.4%, and specificity of 81.8%. The radiomic nomogram had good calibration power.

**Conclusion:**

The radiomic nomogram might has the potential to be used as a non-invasive tool for individual prediction of SPN preoperatively. It might facilitate decision-making and improve the management of SPN in the clinical setting.

## Background

The most common cause of cancer death around the world is the lung and bronchus according to the 2017 cancer statistics [1–3]. Patients with lung cancer usually have a bad prognosis because most of them are diagnosed at an advanced stage (III or IV) with no discriminating symptoms as compared to early stage [4]. In clinical practice, accurate diagnosis of early lung cancer from small pulmonary nodules (SPN) is challenging. The detection of SPN is increasing with years worldwide, mainly because of the wide use of low-dose chest computed tomography (CT) screening. In the Early Lung Cancer Action Project performed by Henschke et al. [5], the detection rate of SPN was as high as 23%, which increased to 39.5% in patients received lung operation [6]. For indeterminate solid and ground-glass nodules, they should be followed with CT at least 2 and 3 years, respectively, according to the international guidelines for the management of SPN [7, 8]. Therefore, accurate diagnosis of SPN using advanced tool will reduce health costs and extensive CT examinations with no additional benefits. Also, clinicians need an non-invasive imaging tool to determine whether a patient needs surgery or long-term follow-up.

Recently, by high throughput extracting quantitative imaging features from standard-of-care medical images, radiomics provides us a promising and non-invasive tool in cancer research [9, 10]. The radiomic features mined by sophisticated bioinformatics tools might involve the process of diagnosis, prognosis and prediction [11]. Radiomic signature constructed by significant features has been applied for precision diagnosis and treatment of cancer, which will promote the development of precision medicine. Currently, radiomics has been used to decode tumor phenotypes, histological subtypes and pathological response of lung cancer [12–14].

Therefore, the aim of this study was develop and validate a radiomic nomogram for the individual preoperative prediction of lung adenocarcinoma from benign SPN, which would improve the decision-making of SPN in clinical practice.

## Methods

### Patients and nodules

**Fig. 1:**
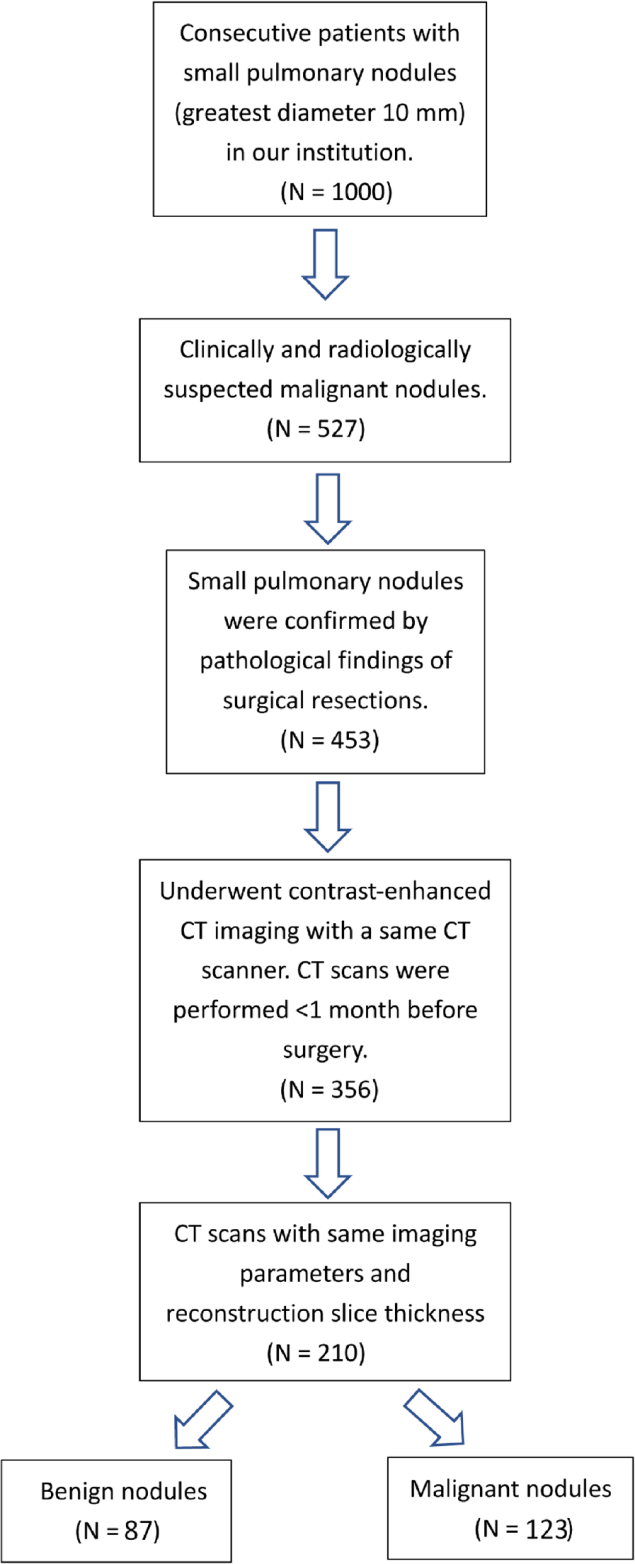
Inclusion pathway of pulmonary nodules

Our institutional review board approved this retrospective study and waived the need for informed consent from patients. A total of 197 patients with 210 SPN treated with surgical resection were included from January 2011 to March 2017. Inclusion criteria were as follows: (1) Patients had histopathologically-confirmed SPN ≤10 mm; (2) Patients had available clinical data; (3) Patients underwent baseline lung CT scan with the same imaging parameters and reconstruction slice thickness; and (4) Patients’ lung CT performed within 1 month before surgery. The patients were excluded if: (1) Patients received surgery before CT scans; and (2) Patients’ lung CT images have breathing artifacts. The patients were randomly divided into training and validation sets by a computer algorithm at a ratio of 7:3. Figure 1 illustrates the study inclusion pathway.

A total of 11 CT findings of each nodule were collected from the last CT scan before surgery, including the maximum diameter, location, involvement of pleura (pleural indentation with or without pleural thickness, absence), nodule consistency (ground-glass nodule [GGN], solid, part-solid GGN), shape (regular [e.g., round, oval] or irregular), margins (lobulation, spiculation, both, absence), cavity (presence or absence), calcification (presence or absence), intranodular changes (necrosis, consolidation, vacuoles, air bronchogram, absence), bronchial disruption (presence, absence, unclear), and vessel convergence sign (presence or absence). Two radiologists with 13 years and 18 years of clinical experience in lung cancer reviewed all of the CT images and reached a consensus.

### Imaging acquisition

Contrast-enhanced CT images were obtained by a 64-slice CT scanner (Siemens Definition AS + 128, Forchheim, Germany). The imaging parameters were as follows: 120 kV; 120 mA; rotation time = 0.5 s; detector collimation = 64 × 0.625 mm; the field of view = 500 mm; and matrix size, 512 × 512. All patients received intravenous administration of iodinated contrast agent (1–1.1 ml/Kg, Ultravist 370, Bayer Pharma AG, Berlin, Germany). The CT images were obtained after a 30 s delay and reconstructed with a slice thickness of 2 mm.

### CT-based radiomic feature extraction and selection

**Fig. 2:**
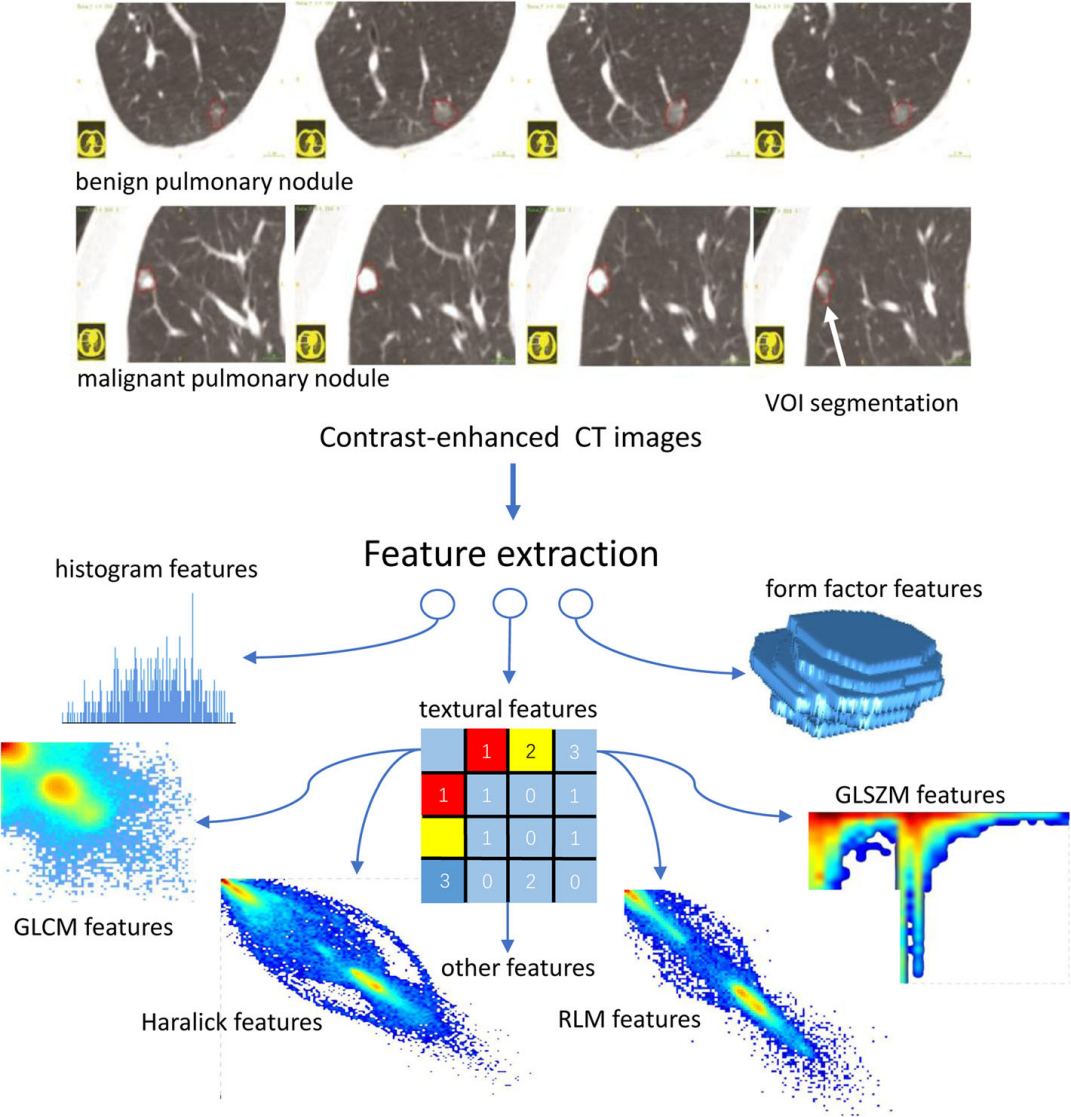
Radiomic workflow. Contrast-enhanced chest CT images are retrieved for radiomic feature extraction. ROIs of pulmonary nodules are segmented and the corresponding ROIs are stacked up to construct VOI of the nodules. Six categories of radiomic features are extracted from within the defined VOI, including histogram features, form factor features, and texture features

Figure 2 shows the radiomic workflow of this study. The regions of interest (ROIs) of pulmonary nodules were delineated by a junior radiologist using open-source ITK-SNAP software (www.itk-snap.org) and validated by a senior radiologist. Radiomic features were extracted from contrast-enhanced CT images by using an in-house feature extraction algorithm applied in Artificial Intelligence Kit software that developed by GE Healthcare Life Sciences. It can be combined with ITK-SNAP software to obtain three dimensional images. A total of 385 radiomic features consisting of form factor features, histogram features, and textural features (such as Gray Level Size Zone Matrix [GLSZM], Gray Level Run Length Matrix [GLRLM], and Gray Level Cooccurrence Matrix [GLCM]). The description of feature extraction algorithms are presented in Supplementary Material.

We applied the least absolute shrinkage and selection operator (LASSO) regression to select the most significant features suggestive malignancy [15]. We performed 100 iterations of 10-fold cross-validation with minimal binomial deviance to select the optimal parameters in LASSO regression [16].

### Training and validation of the conventional CT signature, radiomic signature and radiomic nomogram

To determine the additional value of radiomic signature to conventional CT features, we developed and compared three models (i.e., conventional CT signature, radiomic signature and radiomic nomogram). Conventional CT signature was built based on the results of multivariate logistic regression analysis of 11 conventional CT features. Radiomic signature or radiomic score (Rad-score) was calculated by linearly fitting the selected radiomic features after weighted by their respective coefficients. Finally, radiomic nomogram was constructed by a multiple logistic regression using the selected conventional CT features and Rad-score.

The area under the receiver operating characteristic curve (AUC), accuracy, sensitivity, and specificity were used to evaluate the performance of the three models in the validation dataset. Calibration curve and the Hosmer-Lemeshow test were used to assess the calibration and goodness-of-fit of the radiomic nomogram [17].

### Statistical analysis

All the statistical analyses were performed using R software (version 3.4.2). The packages were used as follows: “glmnet” for LASSO logistic regression, “rms” for nomogram and calibration plots, and “vcdExtra” for Hosmer-Lemeshow test. Differences of patient and nodule characteristics between the training dataset and validation dataset were compared using Chi-square test or Fisher’s exact test or Mann–Whitney U test, if appropriate. The AUC comparison of different models used Delong test. A *P* < 0.05 was considered significant.

## Results

### Patient and nodule characteristics

Table 1 shows patient and nodule characteristics. The mean age of 197 patients was 51.0 years. Of the 210 nodules, 87 (41.4%) were classified as benign, including tuberculomas (15/87, 17.2%), fibrous nodules (13/87, 14.9%), lymph nodes (11/87, 12.6%), hamartomas (13/87, 14.9%), pulmonary cryptococcosis (10/87, 11.5%), inflammatory nodules (8/87, 9.2%), inflammatory granuloma (4/87, 4.6%), aspergillosis (3/87, 3.4%), sclerosing hemangiomas (2/87, 2.3%), and atypical adenomatous hyperplasia (8/87, 9.2%); 123 (58.6%) were malignant, composed of invasive adenocarcinomas (44/123, 35.8%), minimally invasive adenocarcinoma (59/123, 48.0%), and adenocarcinoma in situ (20/123, 16.3%). No significant difference was found between the training and validation datasets in regard to most clinical and imaging features (Table 1).

**Table 1:** Baseline characteristics of patients and nodules

Characteristics	Training dataset (***n*** = 147)	Validation dataset (***n*** = 63)	p
**Age (years)**	51.9 ± 11.3	49.0 ± 11.0	0.079
**Sex**			
Male	60 (40.8%)	16 (25.4%)	0.041
Female	87 (59.2%)	47 (74.6%)	
**Pathological results**			
Benign	53 (36.1%)	24 (38.1%)	0.876
Malignant	94 (63.9%)	39 (61.9%)	
**Maximum Diameter (mm)**	7.8 ± 1.8	7.5 ± 1.9	0.273
**Location**			
Left-upper lobe	43 (29.3%)	14 (22.2%)	0.301
Left-lower lobe	19 (12.9%)	14 (22.2%)	
Right-upper lobe	48 (32.7%)	23 (36.5%)	
Right-middle lobe	13 (8.8%)	6 (9.5%)	
Right-lower lobe	24 (16.3%)	6 (9.5%)	
**Involvement of pleura**^a^			
Pleural indentation without pleural thickness	25 (17.0%)	10 (15.9%)	0.967
Pleural indentation with pleural thickness	4 (2.7%)	2 (3.2%)	
Absence	118 (80.3%)	51 (81.0%)	
**Nodule consistency**			
GGN	45 (30.6%)	23 (36.5%)	0.565
Solid	60 (40.8%)	26 (41.3%)	
Part-solid GGN	42 (28.6%)	14 (22.2%)	
**Shape**^a^			
Regular	141 (98.6%)	61 (96.8%)	1.000
Irregular	6 (4.1%)	2 (3.2%)	
**Margins**			
Lobulation	10 (6.8%)	4 (6.3%)	0.546
Spiculation	27 (18.4%)	5 (7.9%)	
Both	15 (10.2%)	4 (6.3%)	
Absence	95 (64.6%)	50 (79.4%)	
**Cavity**^a^			
Presence	3 (2.0%)	0 (0%)	0.556
Absence	144 (98.0%)	63 (100%)	
**Calcification**			
Presence	3 (1.9%)	0 (0%)	0.556
Absence	144 (100%)	63 (100%)	
**Intranodular changes**^a^			
Necrosis	0 (0%)	0 (0%)	0.177
Consolidation	0 (0%)	0 (3.2%)	
Vacuoles	15 (10.2%)	2 (3.2%)	
Air bronchogram	5 (3.4%)	2 (3.2%)	
Absence	127 (86.4%)	59 (93.7%)	
**Bronchial disruption**^a^			
Presence	0 (0%)	0 (0%)	0.300
Absence	147 (100%)	62 (98.4%)	
Unclear	0 (0%)	1 (1.6%)	
**Vessel convergence sign**			
Presence	0 (0%)	0 (0%)	1.000
Absence	147 (100%)	63 (100%)	

^a^Fisher’s exact test

### Feature selection and radiomic signature construction

**Fig. 3:**
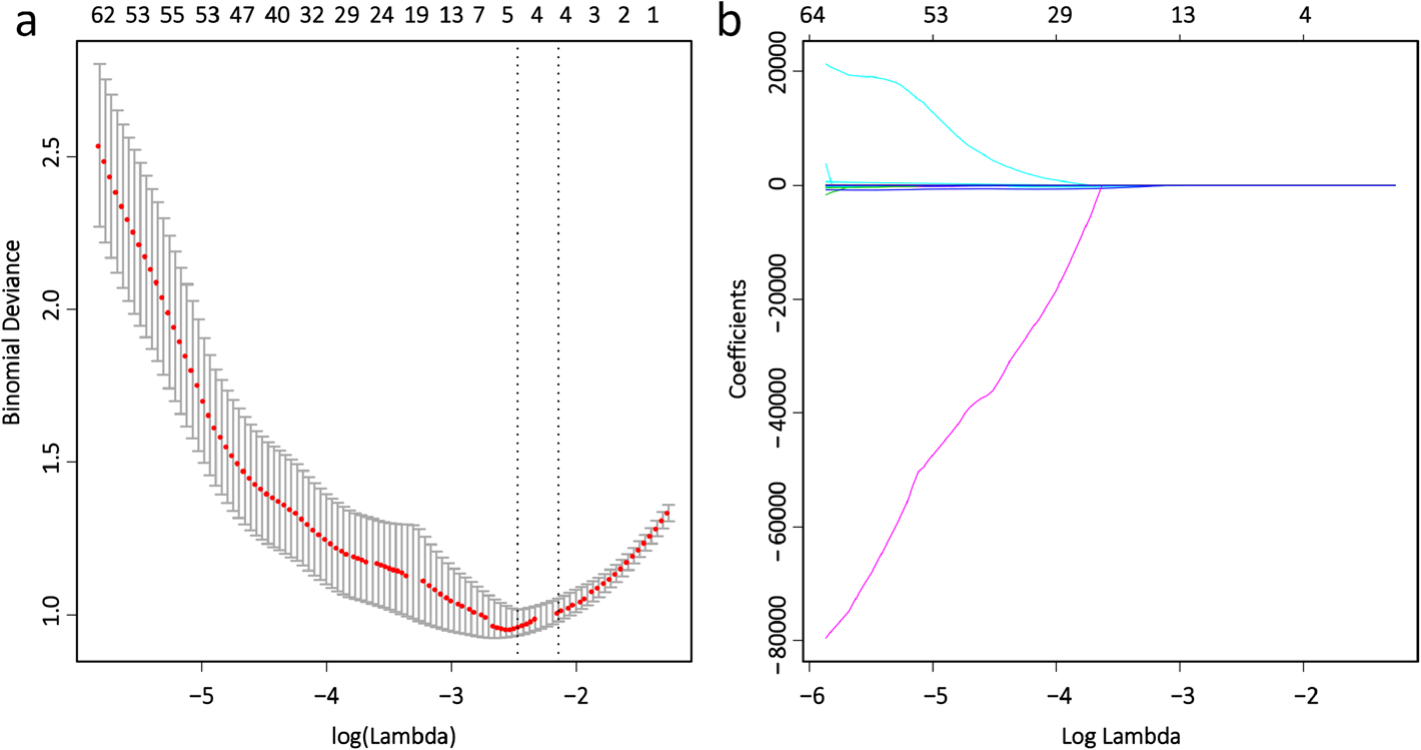
Radiomic feature selection using LASSO logistic regression. **a** Selection of the tuning parameter (λ). The LASSO regression model was used with penalty parameter tuning that was conducted by 10-fold cross-validation based on minimum criteria. The binomial deviance was plotted versus log (λ). The dotted vertical lines were plotted at the optimal λ values based on the minimum criteria and 1 standard error of the minimum criteria. The optimal λ value of 0.0809 with log (λ) = −2.515 was selected. **b** LASSO coefficient profiles of the 385 radiomic features. The dotted vertical line was plotted at the λ value of 0.0809, resulting in five nonzero coefficients

A total of 385 radiomic features were extracted from each volume of interest of the nodules on contrast-enhanced CT images. Five features with non-zero coefficients were selected by LASSO (Fig. 3a-b). The radiomic score calculation formula:

Rad-score =

3.608

-4.133e-03*stdDeviation

-0.214*uniformity

-3.082e-08*ClusterProminence_AllDirection_offset1_SD

-1.105e-9*ClusterProminence_angle90_offset1

-6.712e-05*Inertia_angle0_offset4

**Fig. 4:**
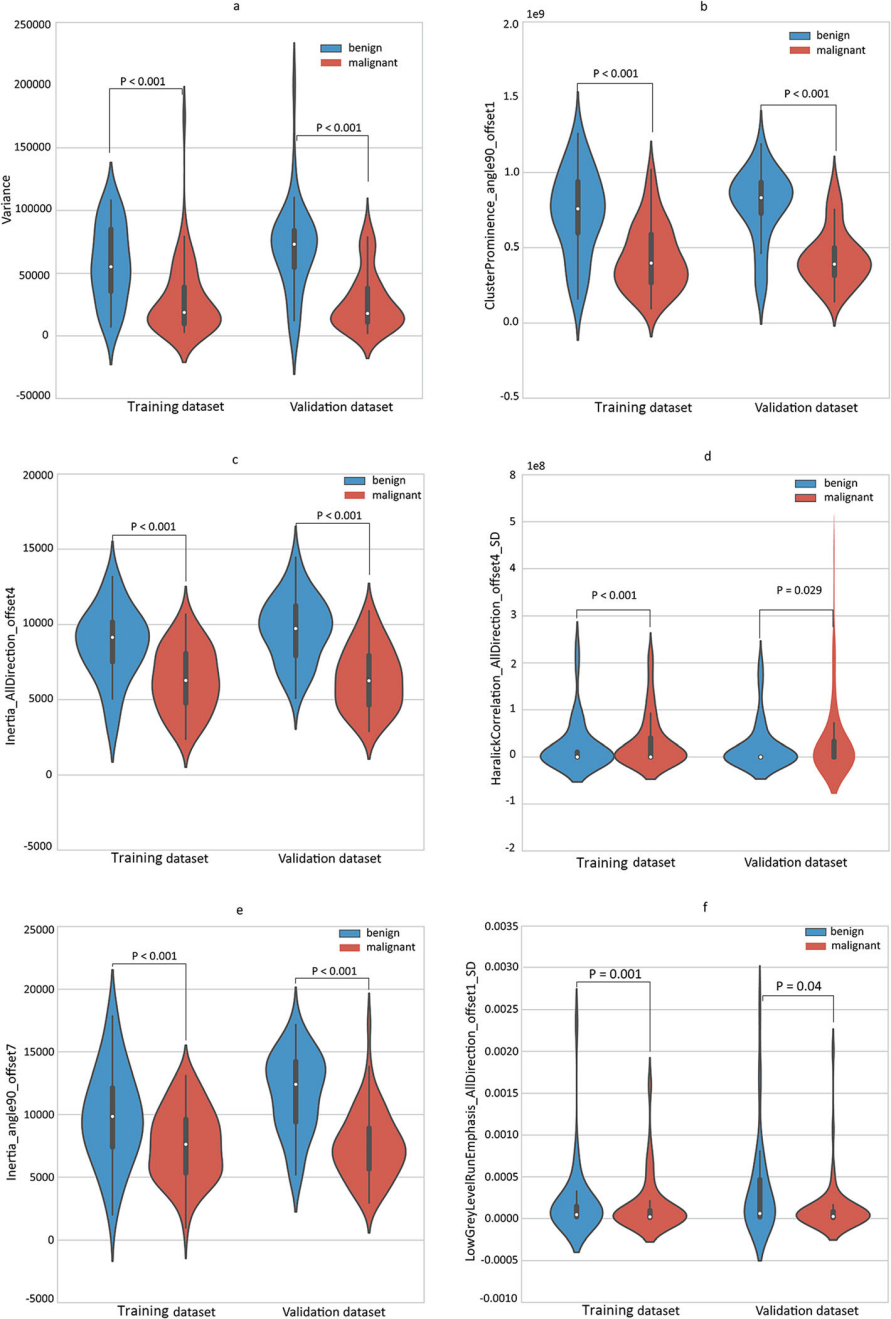
Violin plots present the boxplots of the five radiomic features in the training and validation datasets, respectively

The five radiomic features were significantly different between the benign and malignant SPN (for all, *p* < 0.001) (Fig. 4).

### Training and validation of the conventional CT signature, radiomic signature and radiomic nomogram

**Fig. 5:**
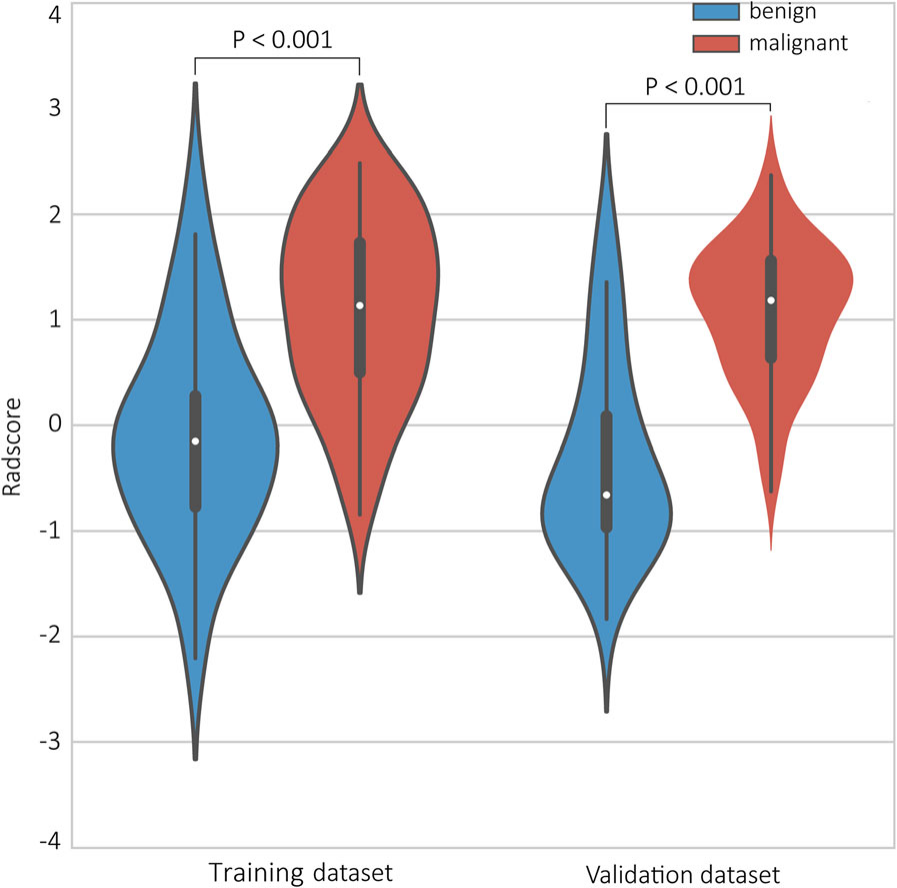
Violin plots present the boxplots of the radiomic score in the training and validation datasets, respectively

The radiomic signature achieved an AUC of 0.878 (95%CI: 0.813 to 0.943), accuracy of 85.0%, sensitivity of 90.1%, and specificity of 76.8% in the training dataset (Table 2) and an AUC of 0.853 (95%CI: 0.735 to 0.970), accuracy of 81.0%, sensitivity of 82.9%, and specificity of 77.3% in the validation dataset (Table 2). The was a significant difference between benign and malignant SPN in regard to Rad-score in the training dataset (median [interquartile range], 1.295 [0.880 to 1.631] vs. -0.525 [− 0.964 to 0.106], respectively, *P* < 0.001, Fig. 5a), which was confirmed in the validation dataset (median [interquartile range], 1.027 [0.444 to 1.841] versus. -0.541 [− 1.208 to − 0.078], respectively, *P* < 0.001, Fig. 5b).

**Table 2:** Predictive performance of clinical-only, radiomics-only, and combined clinical-radiomics models

Models	Training dataset (***N*** = 147)				Validation dataset (***N*** = 63)			
	AUC (95%CI)	Accuracy	Sensitivity	Specificity	AUC (95%CI)	Accuracy	Sensitivity	Specificity
**Conventional CT signature**	0.842 (0.779–0.906)	0.735	62.6%	91.1%	0.833 (0.707–0.958)	65.1%	53.7%	86.4%
**Radiomic signature**	0.878 (0.813–0.943)	0.850	90.1%	76.8%	0.853 (0.735–0.970)	81.0%	82.9%	77.3%
**Radiomic nomogram**	0.911 (0.858–0.965)	0.871	87.9%	85.7%	0.857 (0.723–0.991)	84.1%	85.4%	81.8%

After multivariate analysis, only two CT findings (nodule consistency and margins) remained (*P* < 0.001 and *P* = 0.026, respectively). The two CT features attained an AUC of 0.842 (95%CI: 0.779 to 0.906), accuracy of 73.5%, sensitivity of 62.6%, and specificity of 91.1% in the training dataset and an AUC of 0.833 (95%CI: 0.707 to 0.958), accuracy of 65.1%, sensitivity of 53.7%, and specificity of 86.4% in the validation dataset (Table 2). The AUCs of conventional CT signature and radiomic signature were not significantly different (*P* = 0.292 and 0.586 in the training and validation datasets, respectively).

**Fig. 6:**
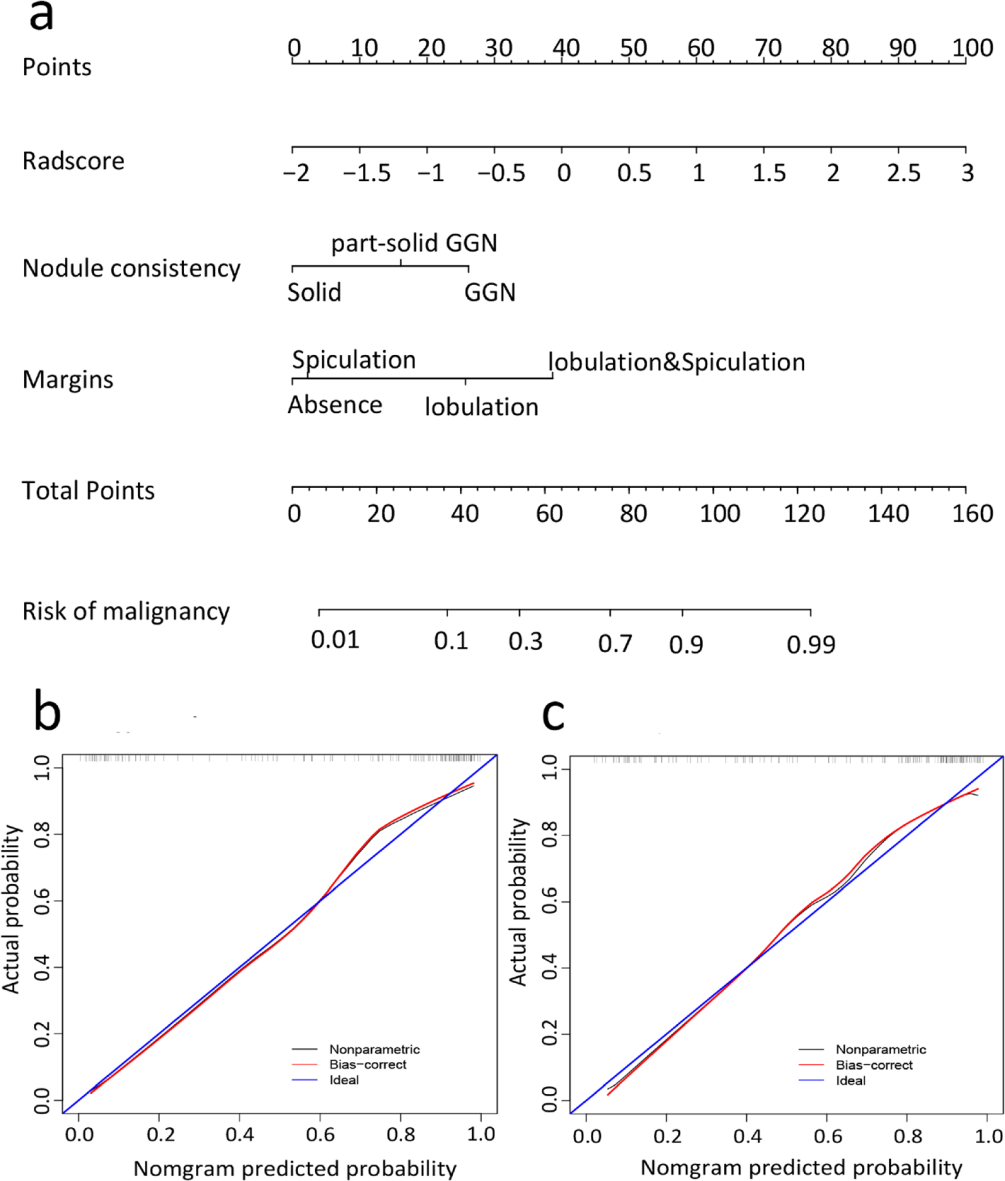
The radiomic nomogram for lung adenocarcinoma prediction. **a** Radiomic nomogram developed for the prediction of lung adenocarcinoma, which incorporates radiomic signature, internal composition and margins of nodule. Plots (**b**) and (**c**) present the calibration curves of the nomogram in the training and validation datasets, respectively. The calibration curve illustrates the calibration of the nomogram in terms of the agreement between the predicted risk of malignancy and the observed outcomes of malignancy. The 45°diagonal line represents a perfect prediction, and the red line represents the predictive performance of the nomogram. The red line has a closer fit to the diagonal line, which indicates better predictive accuracy of the nomogram

A radiomic nomogram incorporating radiomic signature, internal composition and margins of nodule was constructed (Fig. 6a). The radiomic nomogram yielded an AUC of 0.911 (95%CI, 0.858 to 0.965), accuracy of 87.1%, sensitivity of 87.9%, and specificity of 85.7% in the training dataset and an AUC of 0.857 (95%CI: 0.723 to 0.991), accuracy of 84.1%, sensitivity of 85.4%, and specificity of 81.8% in the validation dataset (Table 2), which indicated that the radiomic signature provides added value to the conventional CT features in terms of discriminatory efficacy. The AUC of radiomic nomogram was not significantly different from that of conventional CT features and radiomic signature in the validation dataset (*P* = 0.304 and 0.864, respectively). The calibration curve of the radiomic nomogram is shown in Fig. 6b. The Hosmer-Lemeshow test yielded *P* values of 0.738 and 0.111 in the training and validation datasets, respectively, which indicated good calibration power.

## Discussion

We trained and tested a radiomic nomogram based on the radiomic signature and the anatomical CT features for individualized preoperative prediction of lung adenocarcinoma, which showed good discriminative power and calibration. This study indicates that CT-derived radiomic features supplement the CT findings reported by radiologists in the prediction process. Note that, this study provides a non-invasive and effective prediction tool to determine those patients with a high probability of lung adenocarcinoma.

Early diagnosis of cancer is associated with prolonged survival [18], for instance, the 5-year overall survival of breast cancer was 74.8% between 1975 and 1977; between 2003 and 2009, the number has significantly increased to 90.3% [19]. This increase is mainly due to earlier detection because of the extensive application of mammography for cancer screening [19]. Currently, small pulmonary nodules are still a common and challenging clinical problem. The classification performance of CT is limited, especially in small nodules (≤10 mm in diameter). More accurate and reliable non-invasive diagnostic tool is urgently needed for precise treatment. Early diagnosis of malignant pulmonary nodules is crucial for the improvement of patient’s long-term overall survival.

To date, radiologists diagnose lung cancer by largely depending on qualitative features of CT images, such as nodule diameter, evidence of spiculation, upper lobe location, and pleural indentation [20]. Low-dose CT screening for pulmonary nodules may reduce mortality, however, it also has the risk of overdiagnosis due to detect indolent tumors [5]. Some radiologists contended serial examinations for all serendipitous SPN on CT to render an timely lung operation for cure [7], which may be too aggressive. Excessive detection of SPN might has potential adverse implications on current medical system and clinical practice, such as low utilization of limited resources, raised health care costs, increased radiation and risk for morbidity and mortality of patients [7]. CT-guided percutaneous biopsy has commonly used to obtain tumor histological results due to the characteristics of peripheral location of most pulmonary nodules. However, in actual clinical practice, progressively smaller nodules often result in reduced sensitivity for percutaneous biopsy [21, 22] and other factors also influence the accuracy of biopsy including nodule morphology and length of needle path [20]. In addition, percutaneous biopsy has several limitations, such as invasive nature and high risk for complications [23]. Therefore, non-invasive imaging-based biomarkers are needed to provide additional diagnosis information.

Recently, the increased training of medical image analysis and tools has driven additional studies investigating the radiomics of lung cancer. Radiomic signatures may help to mining bioinformatics behind lung cancer on medical image, for instance, tumor staging [24], gene expression patterns [25], treatment response [26, 27], and patient survival [28, 29]. Current determination of whether radiomic features can improve the prediction of pulmonary nodules as being malignant as opposed to conventional visual assessment on CT is a hot topic [30, 31], but most studies have examined nodules smaller than 30 mm in diameter. In this study, 210 SPN less than 10 mm with surgery-proven malignancy or benign status were included for radiomic analysis. All radiomic features were extracted from a same CT scanner, with same imaging parameters and reconstruction slice thicknesses. As Wu et al. indicated, without control of the variability of factors such as imaging scanners, scanning parameters, the performance of radiomic features could be depressed [32]. An increased number of radiomic features has the potential ability to quantify intra-tumoral heterogeneity. However, most of high-dimensional features are redundant, which will cause poor classification performance. We aimed to select the radiomic features that most associated with lung adenocarcinoma. Only five useful features were selected from 385 features by LASSO algorithm. Unlike previous studies, this study describes some important CT findings that contribute to the differential diagnosis of lung adenocarcinoma. After multivariate analysis, internal composition and margins were two independent clinical features of lung adenocarcinoma. Those nodules with GGN, lobulation and/or signs of speculation had a higher risk for malignancy, which was consistent with the radiologists’ experience. The conventional CT signature attained a accuracy of 0.735 and 0.651 in the training and validation dataset, respectively. We hypothesized that radiomic features could further improve the diagnostic accuracy of a CT signature. Our study demonstrated the predictive performance of conventional CT features was improved by adding radiomic features, attaining accuracy of 0.871 and 0.841 in the training and validation datasets, respectively.

A number of risk models have been developed, of varying complexity for identifying risk of incident lung cancer among patients with visible lung nodules [33–38]. The models were based on significant patient and nodule characteristics. The accuracy and clinical utility of predictive models depends on the case mix of the population in which it was derived and the prevalence of malignancy in that population. The risk prediction models should be externally validated before they are used in a different clinical setting and population. The four validated models were the Mayo Clinic [33], Veterans Administration [34], Herder [39] and Brock [38]. The studies have shown AUC of 0.89 for Mayo Clinic model, 0.74 for Veterans Administration, 0.92 for Herder and 0.90 for Brock. Our radiomic model achieved similar performance, with an AUC of 0.857. Compared with previous models, our model didn’t consider patient data, but included radiomic features extracted from CT images that could reflect intratumoral heterogeneity. However, our model lacks external validation. We hope to explore the added value of radiomics to the existing risk prediction models.

## Conclusions

In summary, this study showed the potential of radiomic features extracted from unenhanced CT images for predicting lung cancer before surgery. Radiomic features showed the added value to the conventional CT features in differentiating lung adenocarcinoma from benign SPN. This study provides doctors a radiomic nomogram as a non-invasive tool for individualized prediction of lung cancer preoperatively. However, before applying in real-world setting, more studies are needed to validate the performance of the radiomic nomogram.

## Supplementary information

**Additional file 1.**

## Data Availability

All data generated or analysed are included in this article.

## Abbreviations

CT: Computed tomography
SPN: Small pulmonary nodules
VOI: Volume of interest
LASSO: Least absolute shrinkage and selection operator
AUC: Area under the receiver operating characteristic curve
CI: Confidence interval
GGN: Ground glass nodule
GLCM: Gray-level co-occurrence matrix
RLM: Grey level run-length matrix
GLSZM: Gray level size zone matrix
