# Supplementary material for: The development and validation of a radiomic nomogram for the preoperative prediction of lung adenocarcinoma
Source: BMC Cancer. 2020 Jun 8;20:533. doi: 10.1186/s12885-020-07017-7 (PMC7278188; doi:10.1186/s12885-020-07017-7)
Supplement: Supplementary file 1 — Additional file 1. [file 12885_2020_7017_MOESM1_ESM.docx]

**Supplementary Material**

**Supplementary S1: Supplementary Methods**

***A detailed description of radiomic features***

In this study, a total of 385 radiomics features were generated from contrast-enhanced CT images. All feature extraction methods were implemented using A.K. (Artificial Intelligence Kit) software. These features were divided into three categories: histogram parameters, form factor features, and texture features.

***Histogram Features***

Histogram parameters are concerned with properties of individual pixels. They describe the distribution of voxel intensities within the CT image through commonly used and basic metrics. The following twenty first order statistics were extracted, such as energy, entropy, maxIntensity, minIntensity, meanValue, mean absolute deviation, medianIntensity, range, root mean square (RMS), standard deviation, stdDeviation Uniformity, variance, volume count, voxelvalue sum, RelativeDeviation, frequency size, quantiles, percentiles, skewness, and kurtosis.

***Form Factor Features***

These group of features includes descriptors of the three-dimensional size and shape of the tumor region. We determined the following shape and size based features: sphericity, surface area, compactness 1, Compactness 2, Maximum 3D diameter, Spherical disproportion, surface-to-volume ratio, volume, volume CC and volume MM.

***Texture Features***

Texture is one of the important characteristics used in identifying objects or regions of interest in an image, texture represents the appearance of the surface and how its elements are distributed. It is considered an important concept in machine vision, in a sense it assists in predicting the feeling of the surface (e.g. smoothness, coarseness…etc.) from image. Various texture analysis approaches tend to represent views of the examined textures form different perspectives, and due to multi-dimensionality of perceived texture, there is not an individual method that can be sufficient for all textures. Therefore, AK software is mainly concerned with texture classification accuracy improvement using textures features statistical based methods.

The radiomic features in the texture parameters mainly consisted of energy, entropy, correlation, inertia, cluster shade, cluster prominence, grey level co-occurrence matrix (GLCM), grey level run-length matrix (RLM), and gray level Size Zone Matrix (GLSZM).

***GLCM Features***

The GLCM represents the joint probability of certain sets of pixels having certain grey-level values. The advantage of the co-occurrence matrix calculations is that the co-occurring pairs of pixels can be spatially related in various orientations with reference to distance and angular spatial relationships, as on considering the relationship between two pixels at a time. As a result, the combination of grey levels and their positions are exhibited apparently. Therefore, it is defined as “a two dimensional histogram of gray levels for pair of pixels, which are separated by a fixed spatial relationship”. However, the matrix is sensitive to rotation. With the change of different offsets define pixel relationships by varying directions.

The rotation angle of an offset: 0°, 45°, 90°, 135° and displacement vectors (distance to the neighbor pixel: 1, 2, 3 ...), different co-occurrence distributions from the same image of reference. GLCM of an image is computed using displacement vector d defined by its radius, (distance or count to the next adjacent neighbor preferably is equal to one) and rotational angles. Following GLCM parameters were extracted in our study: energy of GLCM, entropy of GLCM, Inertia of GLCM, correlation, inverse difference moment and Haralick features. Furthermore, Haralick features including: Haralick correlation, angular second moment, contrast Haralick entropy, HaraVariance, sumAverage, sumVariance, sumEntropy, differenceVariance, differenceEntropy, inversedifferencemoment.

***RLM Features***

The Pr (i,j|θ) of RLM is defined as the numbers of runs with pixels of gray level i and run length j for a given direction θ. RLMs is generated for each sample image segment having directions (0°,45°,90° &135°), then the following 10 statistical features were derived: short run emphasis, long run emphasis, grey level nonuniformity, run length non-uniformity, low grey level run emphasis, high grey level run emphasis, short run low grey level emphasis, short run high grey level emphasis, long run low grey level emphasis and long run high grey level emphasis (1).

***GLZSM Features***

The GLSZM is the starting point of Thibault matrices (2). For a texture image f with N gray levels, it is denoted GSf (s, g) and provides a statistical representation by the estimation of a bivariate conditional probability density function of the image distribution values. It is calculated according to the pioneering Run Length Matrix principle: the value of the matrix GSf (s, g) is equal to the number of zones of size s and of gray level g. The resulting matrix has a fixed number of lines equal to N, the number of gray levels, and a dynamic number of columns, determined by the size of the largest zone as well as the size quantization. The more homogeneous the texture, the wider and flatter the matrix. SZM does not required computation in several directions, contrary to RLM and co-occurrences matrix (COM).

However, it has been empirically proved that the degree of gray level quantization still has an important impact on the texture classification performance. For a general application it is usually required to test several gray level quantization in order to find the optimal one with respect to a training dataset. Empirically, 32 provides often the best result. More precisely, this matrix is particularly efficient to characterize the texture homogeneity, non-periodicity or speckle like texture; it had provided betters characterizations than granulometry (or COM, RLM, etc.) for the classification of cell nuclei, dermis, road quality (bitumen condition) and some textures in PET images (2). The following quantities are defined in AK software: the normalized GLSZM, small zone emphasis, large zone emphasis, gray-level nonuniformity, zone-size nonuniformity, zone percentage, low gray-level zone emphasis, high gray-level zone emphasis, small zone low gray-level emphasis, small zone high gray-level emphasis, large zone low gray-level emphasis, large zone high gray-level emphasis, gray-level variance, zone-size variance.

**References**

1. MM Galloway. Texture analysis using gray level run lengths. Comput Graphics Image Process. 1975;4:172-9.

2. Thibault G, FERTIL B, Navarro C. Texture indexes and gray level size zone matrix application to cell nuclei classification. Pattern Recognit Inf Process. 2009:140-5.
